# The international platform of registered systematic review and meta-analysis protocols (INPLASY) at 3 years: an analysis of 4,658 registered protocols on inplasy.com, platform features, and website statistics

**DOI:** 10.3389/frma.2023.1135853

**Published:** 2023-07-31

**Authors:** João Vitor dos Santos Canellas, Fabio Gamboa Ritto, Alessandro Rodolico, Eugenio Aguglia, Gustavo Vicentis de Oliveira Fernandes, Carlos Marcelo da Silva Figueredo, Mario Vianna Vettore

**Affiliations:** ^1^Department of Research, INPLASY, Inc.—International Platform of Registered Systematic Review and Meta-Analysis Protocols, Middletown, DE, United States; ^2^Department of Oral and Maxillofacial Surgery, College of Dentistry, University of Oklahoma, Oklahoma City, OK, United States; ^3^Department of Clinical and Experimental Medicine, Institute of Psychiatry, University of Catania, Catania, Italy; ^4^Department of Periodontics and Oral Medicine, University of Michigan, Ann Arbor, MI, United States; ^5^School of Medicine and Dentistry, Griffith University, Gold Coast, QLD, Australia; ^6^Department of Health and Nursing Science, University of Agder, Kristiansand, Norway

**Keywords:** INPLASY, systematic reviews, registration, protocols, registry, scoping reviews, evidence synthesis projects

## Abstract

**Background:**

INPLASY^®^ is an international platform for registering systematic reviews and meta-analysis protocols that was launched in March 2020. INPLASY^®^ provides an online database in which the protocols are maintained as permanent public records and can be accessed on its website (www.inplasy.com).

**Methods:**

We described the database features and registered information of all records published since the launch of the registry on March 31, 2023. Additionally, we analyzed the website statistics dataset to explore user experience and promote data transparency.

**Results:**

Four thousand six hundred fifty-eight records were registered in INPLASY^®^, and more than 94% of the protocols were published within 24 h. Most of the submissions were from China, followed by Portugal, Taiwan, Malaysia, and Brazil. The INPLASY^®^ website received 386,395 page views from 64,568 visitors during the first three years. The accesses were obtained from 170 countries. Most of the accesses were from China, followed by the US, the UK, and Portugal. The review status “completed and published” was observed in 898 protocols, and these studies were published in 372 different scientific peer-reviewed journals. The features of INPLASY^®^ include the following: (i) INPLASY^®^ identifier, a unique protocol number; (ii) the digital object identifier (DOI) number, the URL of the protocol linked to a specific DOI; (iii) ORCID update, INPLASY^®^ automatically updates authors' ORCID page, including their protocol; and (iv) search tools, the protocols are freely accessible on www.inplasy.com.

**Conclusions:**

INPLASY^®^ has several practical and useful features that should be considered when planning the registration of a systematic review protocol. Furthermore, the sharp increase in the number of protocols registered in INPLASY^®^ in the first three years and the database statistics demonstrate that INPLASY^®^ has become an important source of systematic review protocols. Therefore, authors should access INPLASY^®^ before planning a future review study to avoid unintended duplication of efforts and to obtain timely registration.

## 1. Background

The registration of systematic review protocols is crucial to avoid duplication of systematic reviews and improve transparency (Straus and Moher, 2010; Dos Santos et al., 2020). The protocol specifies the objectives and methods that will be applied to conduct the review, enabling authors to track what studies are taking place (Chang and Slutsky, 2012). Additionally, discrepancies between the methods described in the protocol and those in the published review can be identified, allowing readers to analyze outcome reporting biases associated with the study. PROSPERO^1^ (Booth et al., 2011) was the first prospective registry for systematic review protocols; however, recently, alternative platforms have become available (Pieper and Rombey, 2022).

INPLASY^®^ is an international publicly accessible platform for registering systematic reviews and meta-analysis protocols, which was officially launched in March 2020^2^ The INPLASY^®^ registry provides an online platform to register systematic review protocols, which are maintained as permanent public records and are free of access on inplasy.com. Although PROSPERO was the first available registry, it seems challenging for a single platform to register all systematic review protocols developed worldwide. Additionally, PROSPERO was supported by the National Institute for Health Research (NIHR), and submissions from the UK were prioritized during the registration process. In contrast, INPLASY^®^ does not prioritize protocols based on the nationality of authors, providing the same registration time for all submissions. Furthermore, INPLASY provides other relevant features, such as a digital object identifier (DOI) number to easily cite each protocol and an automatic update system connecting the protocol with the final published article.

An important difference between PROSPERO and INPLASY is that PROSPERO does not accept systematic reviews without an outcome of clear relevance to human health. Conversely, INPLASY does not impose any such restrictions, and other review projects, such as scoping reviews, are accepted for registration.

We aim to describe the important features of the new international database and summarize the information from all records published in the database since the launch of the INPLASY^®^ registry. Additionally, we collated a website statistics dataset to explore user experience and promote platform transparency.

## 2. Methods

We collected the following data from all protocols published on inplasy.com from its inception until March 31, 2023: (i) the type of review protocol described in the title, such as systematic review, scoping review, overview of reviews, meta-synthesis, mapping review, rapid review, meta-analysis, or network meta-analysis; (ii) study phase at which the protocol was registered (prospective or retrospective registration); (iii) the country of the corresponding author; and (iv) the number of versions of each protocol.

The INPLASY^®^ website statistics were retrieved to quantify the following: the total number of protocols registered by month; the number of user subscriptions; the number of protocols registered by month; the number of user subscriptions by month; the number of website accesses; the number of website accesses by month; the number of protocol views; and the countries of visitors accessing the INPLASY^®^ website.

The review status of all INPLASY^®^ protocols was checked on the website to describe the list of protocols completed and published in peer-reviewed journals. Electronic libraries, including MEDLINE via PubMed, Cochrane Library, and Embase, were examined to identify peer-reviewed protocols containing the INPLASY^®^ unique registration number published in different indexed journals. The search strategy used to find these protocols was [(INPLASY^*^) OR (“International Platform of Registered Systematic Review and Meta-analysis Protocols”)] AND (protocol[Title]). The total number of protocols published as a stand-alone peer-reviewed article registered in INPLASY^®^ was collected.

Finally, the INPLASY^®^ website pages were explored to list the main features of the platform. These included the types of studies accepted for registration, registration requirements, costs, the tracking system version, the platform's funding model, processing time, and the search structure used to locate a record on the INPLASY^®^ website.

## 3. Results

### 3.1. Registration statistics and types of reviews

**Figure 1:**
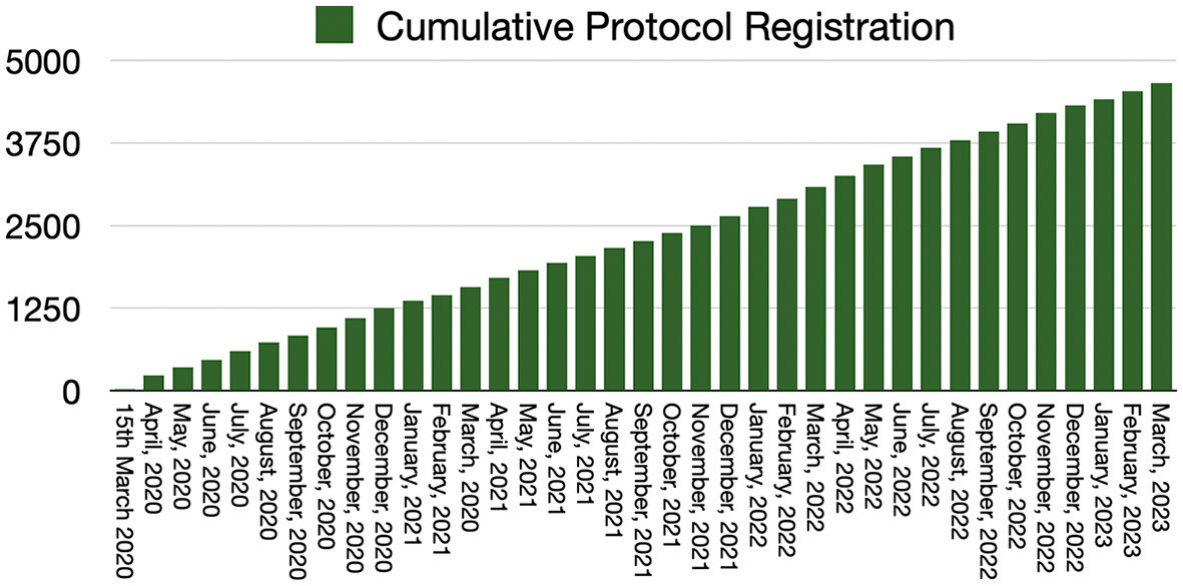
Cumulative total number of INPLASY registrations based on monthly submission rates, 2020–2013.

**Figure 2:**
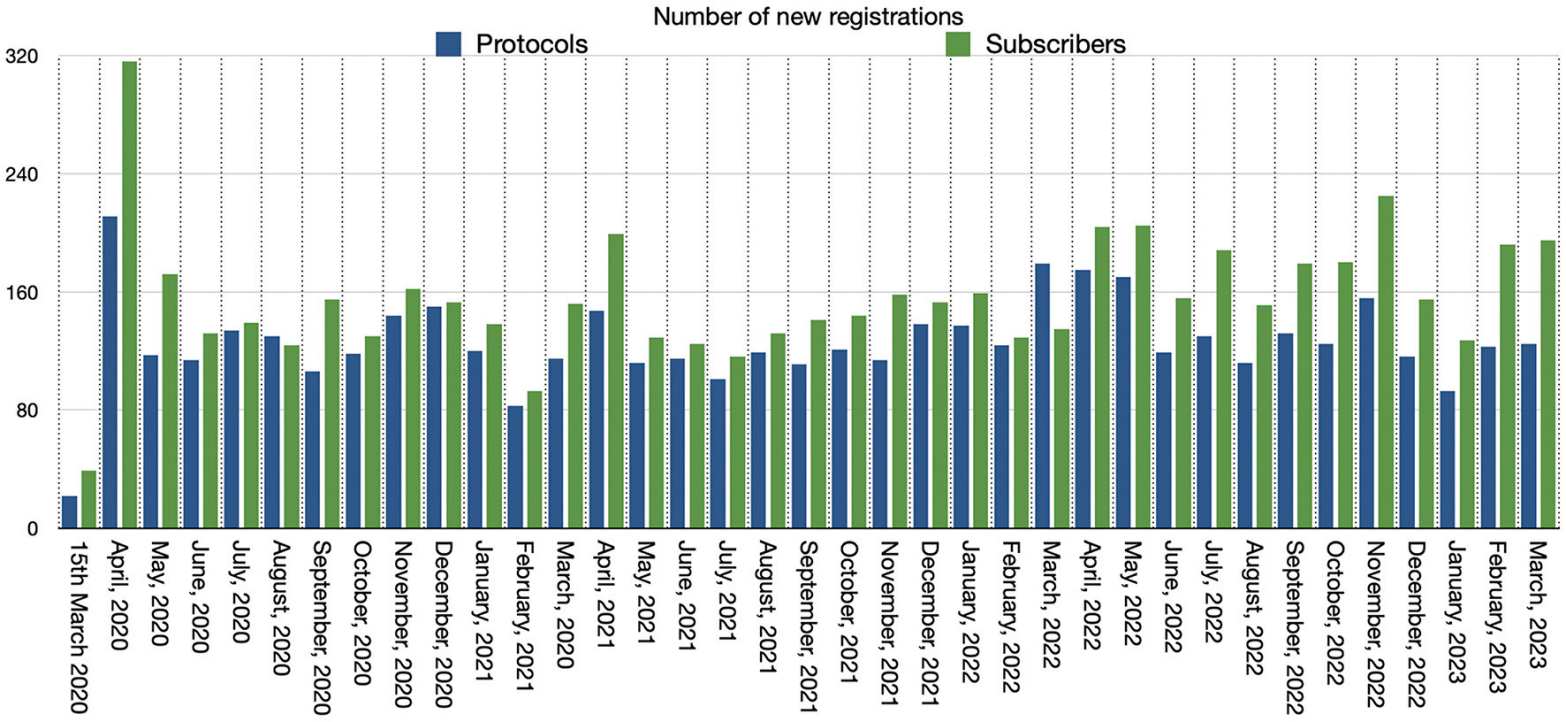
Number of new users and protocols registered monthly in INPLASY.

Four thousand six hundred fifty-eight records were identified in INPLASY^®^ from inception to March 31, 2023 (Figure 1). The number of protocols registered per month is exhibited in Figure 2. On average, ~129 protocols were registered per month. Most submissions were from China, followed by Portugal, Taiwan, Malaysia, and Brazil. There were 5,782 subscribers in March 2023. The number of registered users per month is shown in Figure 2. The registered protocols were from 63 countries where they were developed. Of these, 86.6% were from Asia, 8.0% from Europe, 4.0% from America, 0.62% from Africa, and 0.60% from Australia/Oceania. The types of reviews registered are presented in Table 1. Approximately 80% of registered protocols were obtained from systematic reviews and/or meta-analyses.

**Table 1:** Number of protocols registered in INPLASY^®^ by the types of reviews, from inception to March 31, 2023.

**Type of review (Based on title description)**	**Number of records (Frequency)**
Pairwise systematic reviews and/or meta-analyses	3,701 (79.46%)
Network meta-analysis	461 (9.89%)
Scoping review	117 (2.52%)
Overview of reviews	116 (2.49%)
Diagnostic test accuracy review	40 (0.85%)
Mapping review	10 (0.22%)
Meta-synthesis	8 (0.17%)
rapid review	2 (0.04%)
The title did not specify the type of review	203 (4.36%)
Total	4,658 (100%)

### 3.2. Database statistics

The INPLASY^®^ website received 386,395 page views from 64,568 visitors during the first 3 years. The accesses were from 170 different countries, most of which were from China, followed by the US, the UK, and Portugal.

### 3.3. Registration time and review status

**Figure 3:**
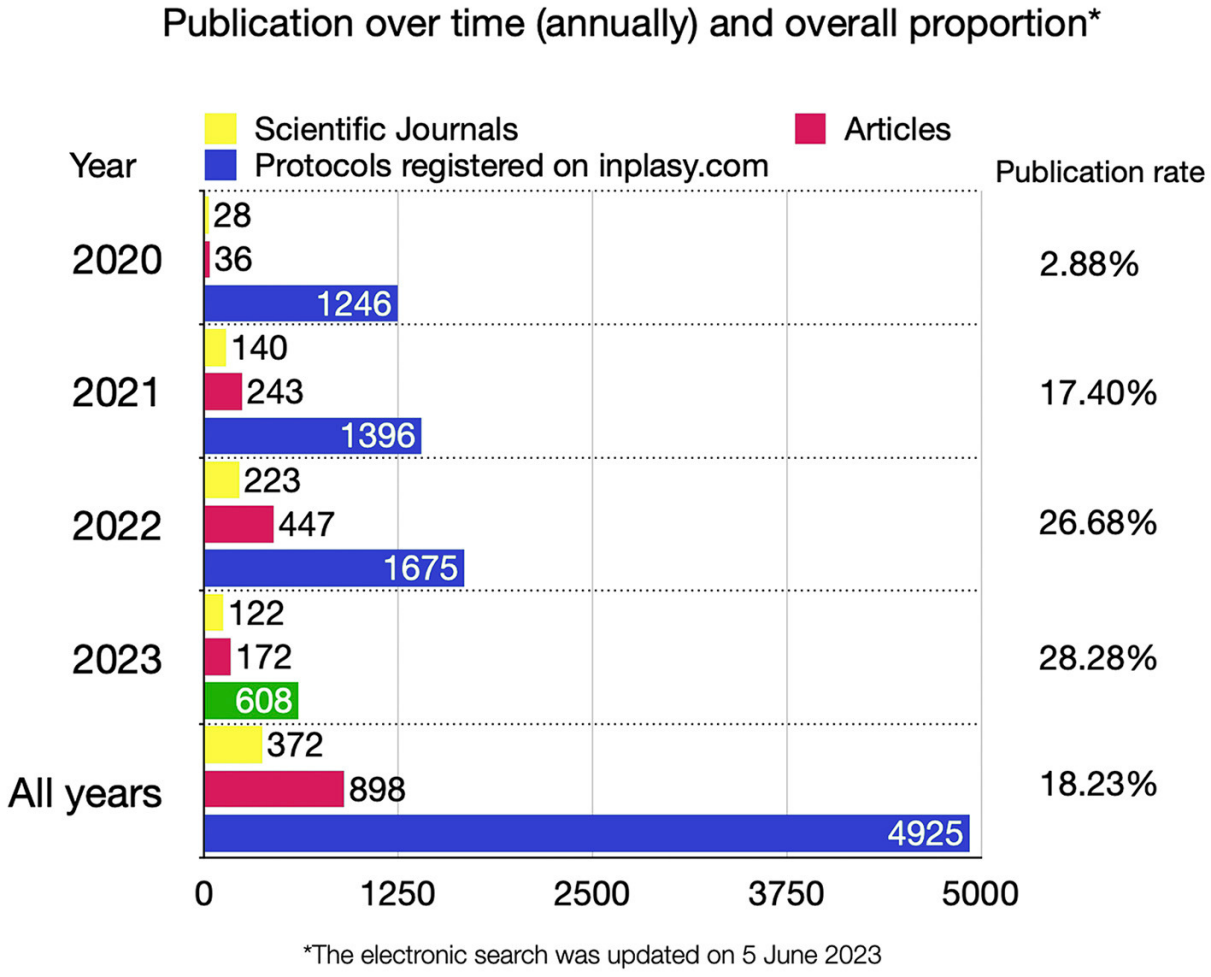
Production of articles and the number of journals containing evidence synthesis projects registered in INPLASY.

Three thousand nine hundred fifty-seven protocols were registered prospectively (84.9%), and 701 protocols (15.1%) were registered retrospectively. The review status “completed and published” was observed in 898 protocols, published in 372 different scientific journals. Figure 3 shows the annual publication rate. Only 0.1% of the INPLASY^®^ protocols presented the updated review status as “discontinued” by authors. Additionally, 692 records registered in INPLASY^®^ were published as stand-alone peer-reviewed protocols (14.85% of the sample), which were published in twelve scientific journals (PLoS ONE, Systematic Review, Medicine (Baltimore), Trials, International Journal of Surgery Protocols, Annals of Palliative Medicine, BMJ Open, European Journal of Integrative Medicine, Evidence-Based Complementary Alternative Medicine, Integrative Medicine Research, and Journal of Pain Research Protocols).

### 3.4. Platform features

#### 3.4.1. Registration number and DOI

INPLASY^®^ provides a unique registration number for each protocol, which can be used to identify the review protocol in the final manuscript. Additionally, all protocols registered on inplasy.com have a DOI, and the URL in which each protocol is hosted is permanently linked to a specific DOI.

#### 3.4.2. Eligible studies

INPLASY^®^ accepts all types of systematic review protocols, including systematic reviews of interventions, diagnostic accuracy, prognostic factors, epidemiological characteristics, and preclinical studies. Systematic reviews assessing sports performance as outcomes are also accepted. Authors can submit scoping review protocols using a standard systematic review form or a specific form developed exclusively for scoping reviews (https://inplasy.com/scoping-reviews/), using the JBI manual for evidence synthesis (Shamseer et al., 2015). One hundred and seventeen scoping review protocols were published in INPLASY^®^ (2.52% of the sample).

#### 3.4.3. Processing time

Four thousand six hundred fifty-eight published protocols were analyzed to determine the period between protocol submission and publication. Ninety percent of the records were published within 24 h, whereas < 1% of the records took over 48 h to be published because of technical issues in the platform or failure during the submission process.

#### 3.4.4. Version tracking—updating a published protocol

Authors can update their protocols using the INPLASY^®^ update form. We identified 4,333 protocols with a single version, 288 protocols with two versions, 29 protocols with three versions, and eight protocols with four versions. All previous versions of the protocol were permanently maintained on the protocol page to allow for a full audit trial in case of any modifications within the record.

#### 3.4.5. Automatic update of the author's ORCID

INPLASY^®^ updates the author's ORCID page using the Crossref interface. Alternatively, authors can manually update their ORCID pages using the DOI number of the protocol. This feature was used in 589 (12.64%) protocols in which at least one of the authors of the registered protocol included ORCID details.

#### 3.4.6. Funding model

INPLASY^®^ was created by a for-profit organization to provide an online public platform on which researchers could register systematic review protocols. The publication fees as of June 2023 were $20 to register a protocol and $9 to update it. During the first 3 years, INPLASY^®^ did not receive funding from government agencies, universities, or other institutions. Therefore, publication fees were the only source of financing during this period. Although a for-profit organization operates the platform, there is an ongoing process involving the public ministry of Distrito Federal Brazil to create a non-profit company for controlling the INPLASY registry. With this transformation, a significant change in the origin of resources for the platform's maintenance is expected by 2023, eliminating any potential conflicts of interest in the for-profit model.

#### 3.4.7. Search structure

The INPLASY^®^ platform offers a simple search tool where records can be found according to the unique identifier number or using free text terms. By June 2023, the Boolean operators (AND, OR, and NOT) could not be used on a search page.

#### 3.4.8. Review process

The INPLASY platform conducts a basic review to certify that a protocol is eligible for registration on the platform. The INPLASY records are not peer-reviewed or assessed for methodological quality. This is the responsibility of authors. The methods or content registered on INPLASY do not constitute an endorsement of methods that are solely those of authors. Additionally, INPLASY does not guarantee the accuracy of the English and is not responsible for errors arising from the text.

## 4. Discussion

Since the launch of INPLASY^®^ in March 2020, the number of records has increased progressively, reaching 4,658 protocols in 63 countries by March 31, 2023. After 3 years of operation, INPLASY^®^ has become the second-largest specific database for the registration of systematic reviews, behind PROSPERO in terms of the number of protocols (Pieper and Rombey, 2022). The INPLASY^®^ protocol was developed based on PRISMA-P recommendations (Shamseer et al., 2015) and the PROSPERO registration form. The INPLASY^®^ form has 33 fields, of which 24 are mandatory and nine are optional. The number of records submitted to PROSPERO over the last 10 years has increased considerably, resulting in an unprecedented number of registrations. Consequently, a significant delay in the registration process was reported before the onset of the COVID-19 pandemic. Puljak (2021) reported waiting for over 6 months to have a protocol published in PROSPERO, which is unacceptable from the author's perspective. PROSPERO implemented a basic automated check system during the pandemic, reducing the waiting time to 30 days. Nonetheless, 30 days are still considered a long waiting period when the registration status is pending, and unawareness of it may result in duplicated efforts.

During the first 3 years, over 95% of the INPLASY^®^ protocols were published in < 24 h. The fast-track processing time of INPLASY^®^ may reduce the duplication of efforts and research waste because the longer the time interval between the submission and the registration, the greater the chances of duplicated protocols. As the platform does not follow a peer-reviewed process, quick registration is possible.

Solla et al. (2020) showed that PROSPERO registration does not prevent two registrations on the same topic. Therefore, authors are responsible for searching for ongoing systematic reviews in the pipeline before submitting their review protocols. COVID-END, a time-limited network group formed by over 50 of the world's leading evidence-synthesis, indicated that before starting a new project, researchers should seek ongoing reviews not only in PROSPERO but also in the INPLASY^®^ platform, the National Collaborating Center, the Center for Evidence-based Medicine, and the VA Evidence Synthesis Program^3^

Other available platforms for the registration of systematic review protocols include Cochrane Reviews, Joanna Briggs Institute, and Campbell Collaboration, which provide quality assurance and many other benefits for accepted review protocols. However, these platforms are highly restricted, and only a small number of selected protocols can be published.

Additionally, these organizations produce only a minority of all systematic reviews (Page et al., 2016). Banno et al. (2022) identified the frequency of systematic review protocols being registered outside the PROSPERO registry until 2019. They listed generic registries that accepted systematic review protocols. Pieper and Rombey (2022) described five alternatives for registering systematic review protocols: PROSPERO, INPLASY, Research Registry, Open Science Framework Registries, and protocols.io. Among them, the first three are specific for systematic review registration. INPLASY^®^ is the only specific systematic review registry that provides a DOI for each protocol. The combination of the unique INPLASY identifier and DOI number allows authors to identify, access, and cite their protocols easily and precisely.

Approximately 85% of the included studies were prospectively registered. Although protocols can be registered retrospectively using INPLASY, the registry does not recommend retrospective registration unless authors explain the reasons preventing prospective registration. The status of the study at the time of submission is registered in the protocol to permit editors, reviewers, and readers to identify which studies were retrospectively registered and evaluate whether retrospective registration was justifiable for each case. Many scientific journals accept retrospectively registered studies because authors disclose this status in a brief statement in the manuscript. Two for-profit organizations operate INPLASY^®^, and on June 2023, all available sources of funds to support the platform were derived from publication fees. During the first 3 years, INPLASY concentrated its marketing activities in Asian countries. Consequently, the platform today has over 85% of records from this geographical area. A wider distribution is expected when the platform switches to a non-profit model because it will be more accessible to different researchers.

Andersen et al. (2021) reported that few authors updated their review status in PROSPERO after publication. Similarly, < 1% of all published reviews registered in INPLASY^®^ were updated by the authors after publication. INPLASY^®^ automatically updates the review status of registered protocols and links them to the URL of the article. Thus, identifying the concluded projects is easier and more straightforward. We identified 372 scientific journals containing reviews registered on the INPLASY^®^ platform. A systematic review published by Li et al. (2023), which was registered in INPLASY^®^, was published in the journal with the highest impact factor (IF) during the first 3 years (The Lancet Gastroenterology and Hepatology, IF = 45.042). The list of all peer-reviewed articles registered in INPLASY^®^ is updated daily and available at https://inplasy.com/published-articles/.

High-quality protocols can be obtained by submitting records to peer-reviewed journals after registration. Several journals, such as PLoS ONE, BMJ Open, and Systematic Reviews, have published systematic review protocols as stand-alone peer-reviewed articles. The advantage is that the methods proposed in the protocol are critically appraised, thereby increasing the quality of the report and preventing potential flaws that may compromise the validity of the study. However, most systematic reviews do not refer to peer-reviewed protocols. Only 14.85% of all protocols registered in INPLASY^®^ were published in peer-reviewed journals, confirming that most systematic review protocols have not yet been peer-reviewed.

## 5. Conclusion

Overall, the present findings indicate that the INPLASY platform has many desirable features and should be considered a reliable and fast platform for registering systematic review protocols.

Additionally, INPLASY^®^ provides an option to register other evidence synthesis protocols, such as scoping, methodological, and rapid reviews. The sharp increase in the number of protocols registered in INPLASY^®^ in the first 3 years and the database statistics demonstrate that it has become an important source of systematic review protocols. Therefore, authors should access INPLASY^®^ before planning a future review study to avoid unintended duplication of efforts and to obtain timely registration.

## Data availability statement

Publicly available datasets were analyzed in this study. This data can be found at: inplasy.com/projects.

## Author contributions

JC: conceptualization, data curation, formal analysis, investigation, writing—original draft preparation, supervision, and finally reviewed the manuscript. FR: data curation, formal analysis, validation, visualization, and writing—original draft preparation. AR and EA: visualization and writing—review and editing the manuscript. GF: data curation, investigation, validation, and writing—review and editing the manuscript. CF: data curation, validation, and writing—review and editing the manuscript. MV: formal analysis, supervision, and writing—review and editing the manuscript. All authors contributed to the article and approved the submitted version.

## Footnotes

^1^PROSPERO International prospective registry of systematic reviews. Available online at: https://wwwcrdyorkacuk/prospero/.

^2^INPLASY International platform of registered systematic review and meta-analysis protocols. Available online at: www.inplasy.com.

^3^COVID-19 Evidence Network to support Decision-making—Resources for researchers—Identify ongoing reviews/avoid duplication effort. Available online at: www.mcmasterforum.org/docs/default-source/covidend/covid-end_researchers.pdf?sfvrsn=437e56d5_4.
